# Revisiting the Endocytosis of the M_2_ Muscarinic Acetylcholine Receptor

**DOI:** 10.3390/membranes5020197

**Published:** 2015-05-12

**Authors:** Wymke Ockenga, Ritva Tikkanen

**Affiliations:** Institute of Biochemistry, Medical Faculty, University of Giessen, Friedrichstrasse 24, D-35392 Giessen, Germany; E-Mail: Wymke.Ockenga@biochemie.med.uni-giessen.de

**Keywords:** acetylcholine, clathrin, dynamin, endocytosis, G protein-coupled receptors, endosomes, flotillins

## Abstract

The agonist-induced endocytosis of the muscarinic acetylcholine receptor M_2_ is different from that of the other members of the muscarinic receptor family. The uptake of the M_2_ receptor involves the adapter proteins of the β-arrestin family and the small GTPase ADP-ribosylation factor 6. However, it has remained inconclusive if M_2_ endocytosis is dependent on clathrin or the large GTPase dynamin. We here show by means of knocking down the clathrin heavy chain that M_2_ uptake upon agonist stimulation requires clathrin. The expression of various dominant-negative dynamin-2 mutants and the use of chemical inhibitors of dynamin function revealed that dynamin expression and membrane localization as such appear to be necessary for M_2_ endocytosis, whereas dynamin GTPase activity is not required for this process. Based on the data from the present and from previous studies, we propose that M_2_ endocytosis takes place by means of an atypical clathrin-mediated pathway that may involve a specific subset of clathrin-coated pits/vesicles.

## 1. Introduction

Muscarinic acetylcholine receptors (mAChRs) belong to the family of G protein-coupled receptors (GPCRs). The five receptor subtypes (M_1_, M_2_, M_3_, M_4_ and M_5_) activate their corresponding G proteins upon ligand binding, after which the receptors are desensitized by three different mechanisms. They can either be inactivated by phosphorylation, which hinders their interaction with G proteins, or they are temporarily internalized and then recycled back to the plasma membrane (reviewed in [1]). Alternatively, the receptors can be downregulated by degradation in lysosomes. Different from the other mAChR subtypes, the M_2_ receptor internalizes slowly, and it is frequently not resensitized, nor does it recycle back to the plasma membrane [2,3,4]. Therefore, degradation in lysosomes seems to be the major pathway of M_2_ desensitization after endocytosis [5].

Among the family of mAChRs, the M_1_, M_3_ and M_5_ receptors have all been shown to undergo endocytosis in a clathrin- and dynamin-dependent manner (see, e.g., [4,6]), whereas M_4_ is apparently internalized by means of a recently described fast endophilin-mediated endocytosis (FEME) [7,8]. However, internalization of the M_2_ receptor appears to be regulated in a somewhat different manner. Proteins that have been suggested to play a role in ligand-induced M_2_ uptake are β-arrestins, the small GTPase Arf6 (ADP-ribosylation factor 6), clathrin and dynamin (reviewed in [1]).

Arrestins are cytosolic clathrin-binding proteins that are associated with mAChR trafficking. The M_2_ receptor is phosphorylated upon agonist stimulation, which is mainly mediated by the G protein-coupled receptor kinase 2 (GRK2), and receptor phosphorylation regulates the interactions with the members of the arrestin protein family [9,10,11,12,13,14]. Although several earlier studies suggested that M_2_ internalization does not depend on β-arrestins [4,9,14], it was later shown in mouse embryonic fibroblasts from β-arrestin-1 and -2 double knockout mice that internalization of the M_2_ receptor was inhibited. However, this phenotype could be rescued upon expression of β-arrestin-1 or -2, demonstrating that these proteins indeed play a role in M_2_ trafficking and endocytosis [15].

The small GTPase Arf6 plays a role in both clathrin-dependent and -independent endocytic processes, but it is also involved in endocytic recycling (reviewed in [1]). Trafficking of the M_2_ receptor is also influenced by Arf6, and the expression of a constitutively active Arf6 mutant impairs endocytosis and/or enhances M_2_ recycling [16,17,18]. It has been suggested that the Arf6-mediated M_2_ uptake takes place by means of a clathrin-independent pathway which involves specific carriers that do not contain markers for the typical clathrin-mediated pathway [18]. However, after the immediate uptake of these carriers, they rapidly merge with early endosomes that also contain the typical clathrin-based cargoes and markers [16].

The greatest controversies in the literature in terms of M_2_ endocytosis are associated with the role of clathrin and dynamin in this process. It has been shown that endocytosis of the M_2_ receptor is not affected by the expression of the clathrin heavy chain (CHC) hub mutant or the amphiphysin B/C domain, which both exert a dominant-negative effect on clathrin-mediated endocytosis [4,19]. This has led to the conclusion that M_2_ is endocytosed in a clathrin-independent manner. On the other hand, hypertonic sucrose, which specifically inhibits clathrin-mediated endocytosis, also prevents M_2_ uptake upon agonist stimulation [5,19]. Thus, the role of clathrin in M_2_ endocytosis has not been conclusively dissected.

Previous findings on the role of the large GTPase dynamin in M_2_ endocytosis are divergent, depending on the experimental setting used. Some studies have concluded that dynamin is not required for M_2_ receptor endocytosis, as the dominant negative K44A dynamin mutant does not show any effect on M_2_ uptake [4,9,12]. However, further studies have used dynamin mutants that impair dynamin function in a different manner, such as the K535M (not stimulatable by phosphatidylinositol 4,5-bisphosphate) or the N272 deletion mutant (lacks the GTP binding domains) [20,21], ending up in the opposite conclusion, according to which dynamin indeed plays a role in M_2_ endocytosis [22]. Thus, it has been suggested that M_2_ endocytosis exhibits an atypical sensitivity to dynamin [19], but the details of this dependency have remained unclear.

Taken together, it is assumed that M_2_ receptor endocytosis takes place by means of a β-arrestin- and Arf6-dependent pathway, whereas the role of dynamin and clathrin has remained inconclusive. Therefore, there is no clear consensus in the literature about the uptake mechanisms of the M_2_ receptor, although the role of β-arrestins and Arf6 appears to be clearly demonstrated. We have here revisited the role of clathrin and dynamin in M_2_ endocytic trafficking, whereas β-arrestins and Arf6 were not a subject of our study. For this purpose, we have applied tools that have only recently become available and have thus not been used by previous studies addressing M_2_ endocytosis. Clathrin function was impaired by means of siRNA-mediated knockdown of the CHC, instead of overexpression of dominant-negative mutants, such as hub. The role of dynamin was addressed not only by means of the expression of various dominant-negative mutants, but also by chemical inhibition of dynamin function. We here show that agonist-induced M_2_ endocytosis is clathrin- and dynamin-dependent in HEK 293T cells. However, our findings reveal that although dynamin as such is necessary for M_2_ uptake, its GTPase activity is not an absolute prerequisite for endocytosis to occur. These data may imply that M_2_ uptake is accomplished by means of a specific subset of clathrin-coated pits that exhibit an atypical requirement for dynamin activity.

## 2. Results and Discussion

### 2.1. Results

#### 2.1.1. Fluorescent Fusion Proteins of M_2_ Receptor Undergo Agonist-Stimulated Endocytosis

To study the cellular trafficking of M_2_ receptors, we generated constructs for fusion proteins of M_2_ with enhanced green fluorescent protein (M_2_-EGFP) and the monomeric red fluorescent protein (M_2_-DsRed). Use of ectopically-expressed receptor chimeras was necessary, as the lack of antibodies that would allow a reliable detection of the endogenous receptor is a well-known problem in this field. Transient overexpression is commonly used to study the internalization of the M_2_ receptor after agonist treatment [2,9,17,22,23]. The receptor is usually expressed in HEK 293 cells that have been shown to exhibit very low levels of endogenous mAChRs [10], making saturation of the trafficking machinery less likely to occur, despite cargo overexpression.

**Figure 1:**
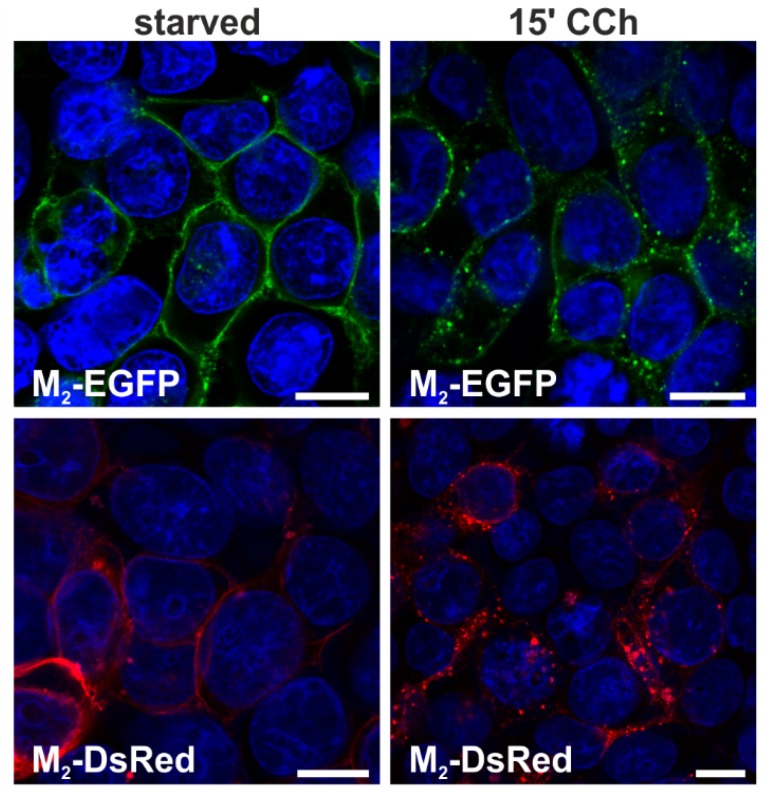
CCh stimulation induces internalization of M_2_-EGFP and M_2_-DsRed in HEK 293T cells. HEK 293T cells were transiently transfected with plasmids coding for M_2_-EGFP or M_2_-DsRed. The cells were starved for 18 h in serum-free medium and either left untreated or stimulated with 1 mM CCh for 15 min. Cells were fixed and the nuclei stained with DAPI (shown in blue). Scale bars: 10 μm.

First, the ability of the M_2_ fluorescent fusion proteins to be internalized after agonist stimulation was analyzed. HEK 293T cells were transiently transfected with either M_2_-EGFP or M_2_-DsRed and starved without serum for 18 h. Both fusion proteins were localized to the plasma membrane in unstimulated cells (Figure 1, left), and after stimulation with 1 mM carbachol (CCh) for 15 min, both receptor chimeras were internalized and localized in intracellular vesicles (Figure 1, right). These data show that our fluorescent M_2_ fusion proteins are normally localized and can undergo agonist-stimulated endocytosis.

#### 2.1.2. Knockdown of Clathrin Heavy Chain Impairs M_2_ Endocytosis

**Figure 2:**
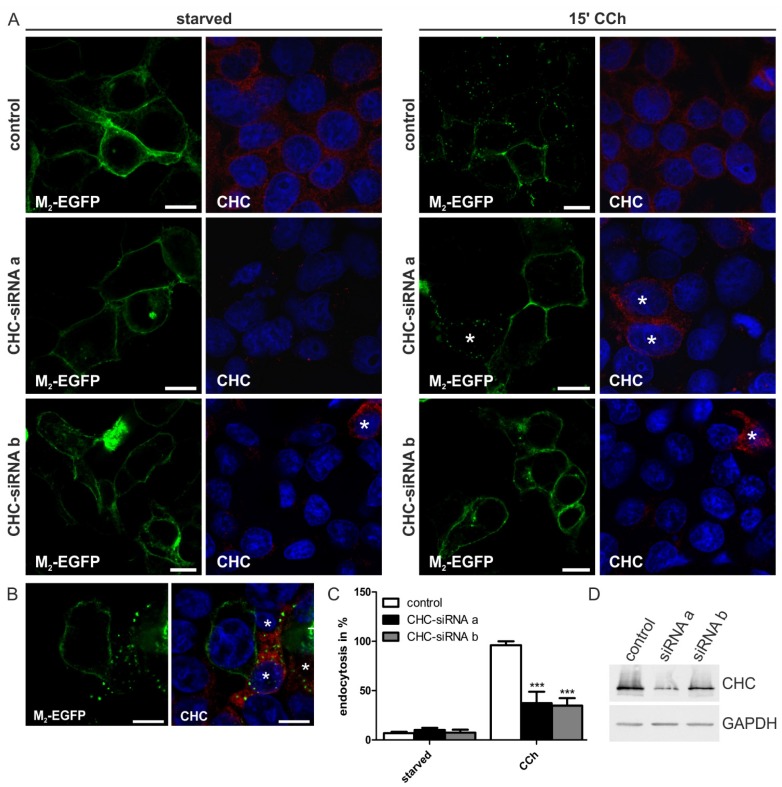
Knockdown of clathrin heavy chain hinders M_2_ receptor internalization. (**A**,**B**) HEK 293T cells were depleted of CHC by two different siRNAs and transfected with M_2_-EGFP. Cells were starved for 18 h in serum-free medium and either left untreated or stimulated with 1 mM CCh for 15 min. Cells were fixed and immunostained for CHC, and the nuclei were stained with DAPI (shown in blue). Scale bars: 10 μm. Single cells still expressing some CHC are marked with an asterisk; (**C**) The number of cells displaying M_2_ receptor internalization was quantified and is shown as the percentage of cells showing intracellular M_2_ localization. At least 100 cells were counted per condition. Results are shown as the mean ± SD. Statistical analysis was performed with two-way ANOVA: *** *p* < 0.001; (**D**) Equal amounts of protein in cell lysates were separated by SDS-PAGE and immunoblotted to monitor CHC knockdown efficiency (about 60%).

Internalization of the M_2_ receptor upon agonist stimulation is generally assumed to be clathrin-independent, as it is insensitive to the expression of the CHC hub mutant and to dynamin-2 K44A mutant expression. The CHC hub mutant is a dominant-negative form that blocks the classical clathrin-mediated endocytosis [24], but not M_2_ receptor uptake [19]. On the other hand, there is some evidence that clathrin might be involved in M_2_ internalization, as M_2_ endocytosis can be inhibited by hypertonic sucrose [5,19]. Furthermore, mouse embryonic fibroblasts from β-arrestin 1/2 double-knockout mice are not able to internalize the M_2_ receptor upon agonist stimulation, and this phenotype is only modestly rescued upon the ectopic expression of arrestins defective in clathrin interaction [15,19,25]. To clarify the role of clathrin in the endocytosis of the M_2_ receptor, the influence of CHC depletion by means of two different siRNAs was studied in HEK 293T cells transfected with M_2_-EGFP (Figure 2). Depletion of CHC with either of the two siRNAs did not change the localization of M_2_ to the plasma membrane in starved cells (Figure 2A, left). However, when the cells were stimulated with CCh, the M_2_ receptor remained at the plasma membrane in clathrin-depleted cells and was only poorly internalized (Figure 2A, right). The fraction of cells showing receptor endocytosis was significantly reduced to about 40% in CHC siRNA transfected cells, as compared to over 95% in control cells (Figure 2C). The CHC siRNAs resulted in a moderate depletion of CHC in Western blot analysis (Figure 2D). However, a closer look at immunostained cells revealed that within the depleted population, cells still expressing some CHC (marked with an asterisk in Figure 2A,B) were capable of receptor internalization, whereas cells devoid of CHC were not. Thus, clathrin appears to be required for M_2_ receptor uptake from the plasma membrane.

#### 2.1.3. M_2_ Endocytosis Is Independent of Flotillins

**Figure 3:**
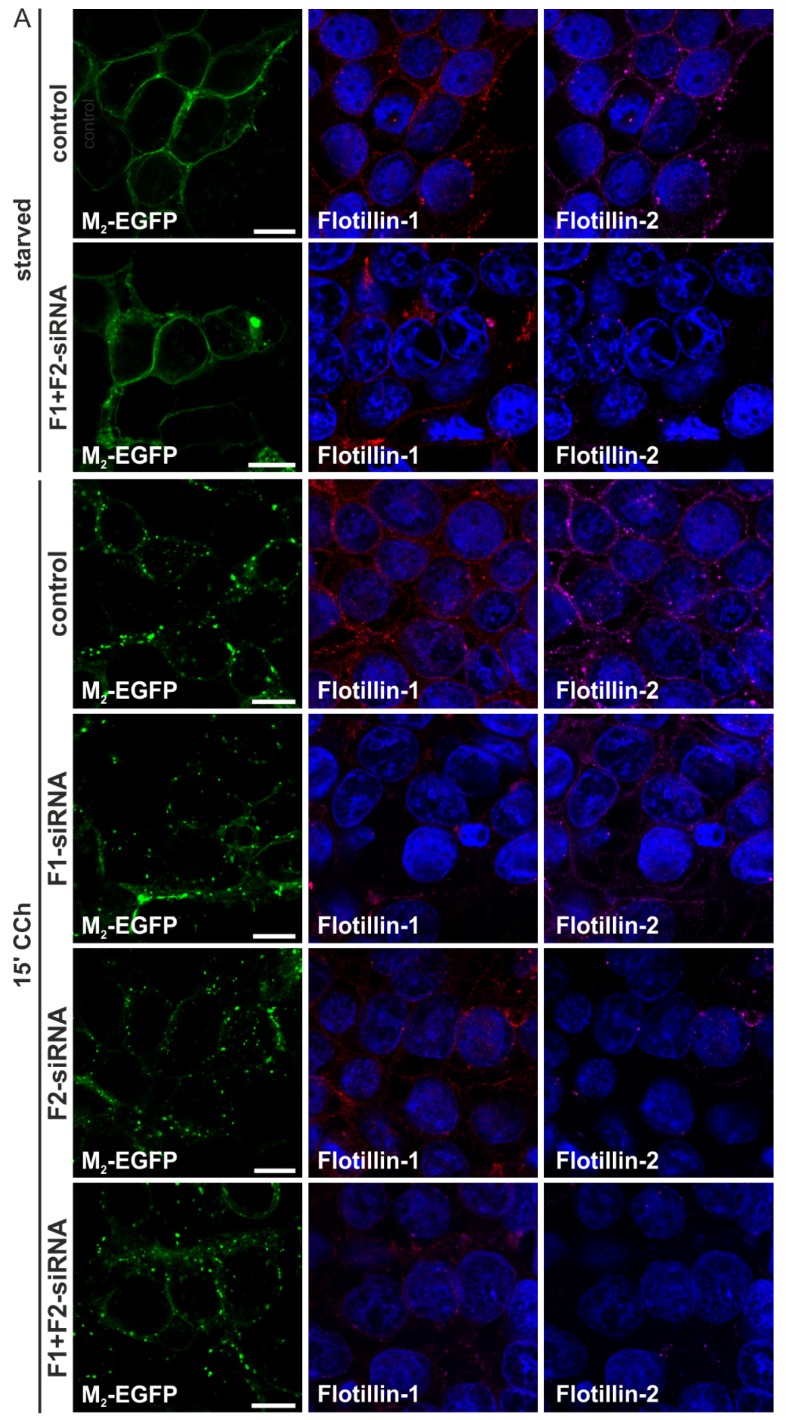

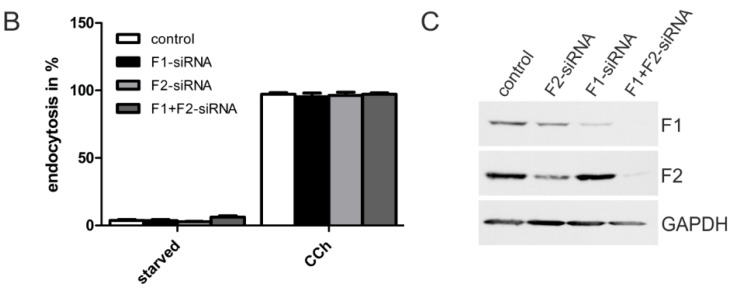
Knockdown of flotillin-1 and flotillin-2 has no impact on M_2_ receptor internalization. (**A**) HEK 293T cells were depleted of flotillin-1 (F1), flotillin-2 (F2) or both (F1 + F2) by specific siRNAs and transfected with M_2_-EGFP. The cells were starved for 18 h in serum-free medium and either left untreated or stimulated with 1 mM CCh for 15 min. Cells were fixed and immunostained for flotillin-1 and flotillin-2. The nuclei were stained with DAPI (shown in blue). Scale bars: 10 μm; (**B**) The fraction of cells displaying M_2_ receptor internalization was quantified and is shown as the percent of cells showing intracellular M_2_ localization. At least 100 cells were counted per condition. Results are shown as the mean ± SD. Statistical analysis was performed with two-way ANOVA; (**C**) Equal amounts of cell lysates were separated by SDS-PAGE and immunoblotted to monitor the knockdown efficiency.

Flotillins, especially flotillin-1, are associated with membrane trafficking processes, including endocytosis (reviewed in [26], see also [27,28]). Our recent data show that flotillin-1 is involved in mAChR signaling in keratinocytes [29], but no data exist on the possible role of flotillins in mAChR endocytosis. Since CHC knockdown did not completely prevent M_2_ endocytosis, we tested if flotillins might be involved. To study this, knockdown experiments were performed with siRNAs against either or both flotillins. Reduction of flotillin levels had no influence on the M_2_ receptor localization in starved HEK 293T cells (Figure 3A, shown for control siRNA and double-depleted cells), and even in agonist-treated cells, depletion of one or both flotillins did not impair M_2_ internalization (Figure 3A). The fraction of flotillin-depleted cells showing M_2_ endocytosis was equal to the controls (Figure 3B). The degree of flotillin depletion was monitored by Western blot (Figure 3C). Please note that flotillin-2 depletion also impairs flotillin-1 expression, due to the reduced stability of the protein. These data show that flotillins do not play a role in the uptake of the M_2_ receptor from the plasma membrane.

#### 2.1.4. Dynamin-2 Mutants and Inhibitors Display Different Effects on M_2_ Endocytosis

There are contradictory findings in the literature when it comes to the involvement of the large GTPase dynamin in M_2_ receptor endocytosis. Traditionally, the effect of dynamin on the endocytosis of specific cargo proteins has been analyzed by expressing functionally-impaired forms of dynamin and checking their effect on cargo endocytosis. In the last few years, chemical inhibitors of dynamin function have become available, but they have so far not been tested in M_2_ receptor internalization. We here analyzed the effect of both dynamin-2 mutants and dynamin inhibitors in agonist-induced M_2_ uptake.

**Figure 4:**
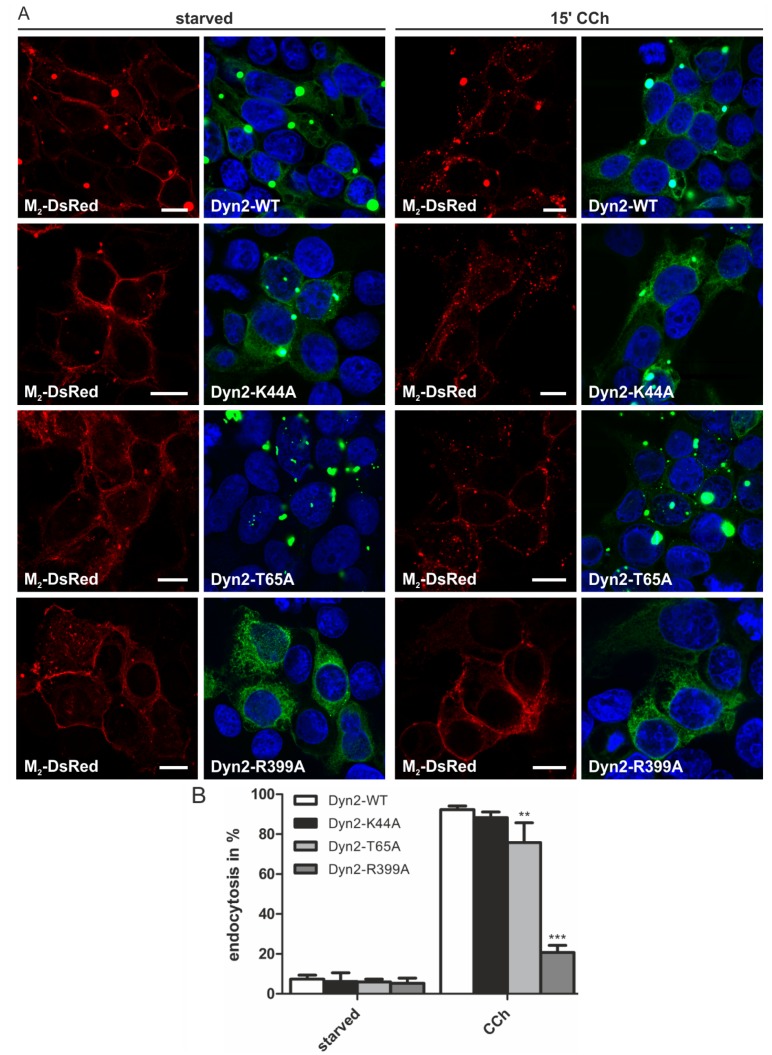
Dynamin mutants have different effects on the agonist-mediated M_2_ endocytosis. (**A**) HEK 293T cells were transfected with M_2_-DsRed and the indicated dynamin-2-EGFP constructs. The cells were starved for 18 h in serum-free medium and either left untreated or stimulated with 1 mM CCh for 15 min. Cells were fixed, and the nuclei were stained with DAPI (shown in blue). Scale bars: 10 μm; (**B**) The fraction of cells displaying M_2_ receptor internalization was quantified and is shown as the percentage of cells exhibiting intracellular M_2_ localization. At least 100 cells were counted per condition. Results are shown as the mean ± SD. Statistical analysis was performed with two-way ANOVA: ** *p* < 0.01; *** *p* < 0.001.

To study the role of dynamin in M_2_ endocytosis, wild-type (WT) and various dominant-negative mutant forms of dynamin-2 were expressed as EGFP fusions. The dynamin-2 K44A mutant is impaired in its GTP binding capacity and, thus, exhibits lower GTP hydrolysis [30], whereas the T65A mutant is able to bind GTP, but is defective in the GTPase activity [31]. The R399A substitution leads to an impaired self-assembly and membrane localization of dynamin-2 [32]. The dynamin-2-EGFP constructs were cotransfected with M_2_-DsRed fusions in HEK 293T cells. The WT dynamin-2 and some of the mutants exhibited a very high level of expression and tended to form aggregates that possessed a very high fluorescence, and a corresponding signal was also detectable in the red channel for most of these aggregates (Figure 4A, left). This may indicate that the M_2_ receptor co-aggregated together with dynamin in these spots. However, except for these unspecific dotty signals that may also partially result from signal overflow, the M_2_-DsRed fusion protein was localized at the plasma membrane in starved cells. None of the expressed dynamin-2 mutants altered the M_2_ localization in starved HEK 293T cells. Expression of dynamin-2 WT or K44A did not prevent M_2_ receptor endocytosis in CCh-stimulated cells, whereas T65A produced a mild reduction in the fraction of cells exhibiting M_2_ endocytosis (Figure 4A, right, and 4B). However, in cells expressing the R399A mutant, M_2_ endocytosis was significantly impaired, and only some 20% of the cells showed M_2_ endocytosis, as compared to 92% of WT-expressing cells (Figure 4B).

**Figure 5:**
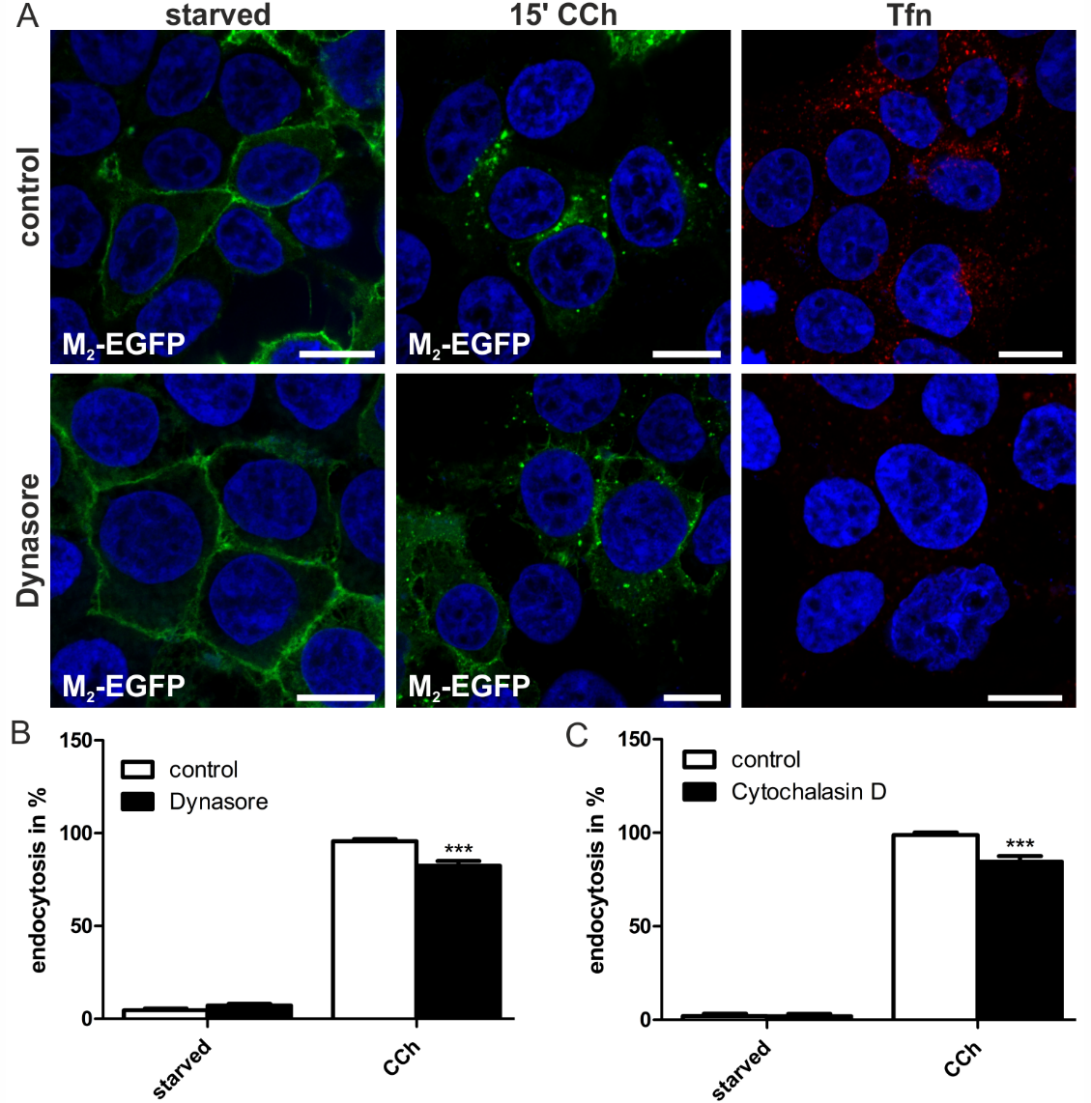
Dynasore moderately reduces CCh-induced M_2_ endocytosis. (**A**) HEK 293T cells were transiently transfected with M_2_-EGFP, starved for 18 h in serum-free medium and pretreated with 80 μM Dynasore or DMSO. Cells were left untreated, stimulated with 1 mM CCh for 15 min or incubated with Alexa Fluor 546-coupled transferrin (Tfn546, red); Cells were fixed, and the nuclei were stained with DAPI (shown in blue). Scale bars: 10 μm. (**B**) The number of cells displaying M_2_ receptor internalization was quantified and is shown as the percentage of cells exhibiting intracellular M_2_. At least 100 cells were counted per condition. Results are shown as the mean ± SD. Statistical analysis was performed with two-way ANOVA: *** *p* < 0.001; (**C**) Quantification of M_2_-EGFP endocytosis in cytochalasin D treated cells.

To verify the data obtained upon expression of the dominant-negative dynamin-2 mutants, two commercially available pharmacological dynamin inhibitors were employed. Dynasore is a cell-permeable substance that reversibly inhibits dynamin GTPase activity and is expected to block dynamin-dependent vesicle formation at the plasma membrane [33,34], whereas MiTMAB interferes with the membrane association of dynamin [35]. Interestingly, although Dynasore completely blocked the uptake of fluorescently-labeled transferrin, a classical cargo of dynamin-mediated endocytosis, it only exhibited a mild, albeit significant, inhibitory effect on M_2_ uptake (Figure 5A,B). Since Dynasore has been described to display unspecific effects on actin [36], we tested if disruption of the actin cytoskeleton would inhibit M_2_ uptake. Indeed, a similar modest but significant inhibition of M_2_ endocytosis was observed upon cytochalasin D (Figure 5C) and latrunculin A (data not shown) treatment. These data thus suggest that the inhibitory effect of Dynasore on M_2_ endocytosis might not be due to direct impairment of dynamin activity, but due to unspecific effects that it displays on actin cytoskeleton.

**Figure 6:**
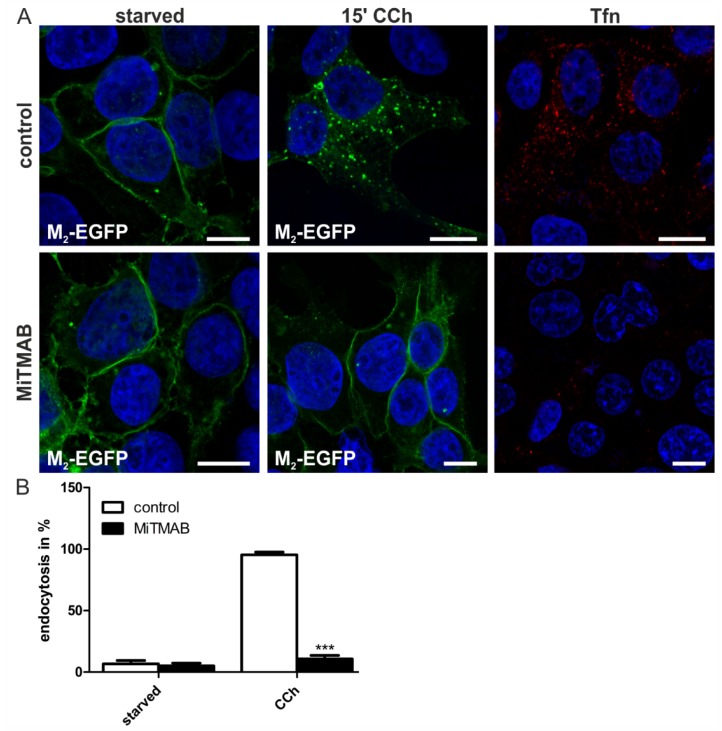
MiTMAB efficiently inhibits CCh-induced M_2_ endocytosis. (**A**) HEK 293T cells were transfected with M_2_-EGFP, starved for 18 h in serum-free medium and pretreated with 30 μM MiTMAB. Cells were left untreated, stimulated with 1 mM CCh for 15 min or incubated with Tfn546. Cells were fixed, and the nuclei were stained with DAPI (shown in blue). Scale bars: 10 μm; (**B**) The fraction of cells displaying M_2_ receptor internalization was quantified and is shown as the percentage of cells exhibiting intracellular M_2_. At least 100 cells were counted per condition. Results are shown as the mean ± SD. Statistical analysis was performed with two-way ANOVA: *** *p* < 0.001.

In contrast to Dynasore which inhibits dynamin GTPase activity, MiTMAB prevents dynamin membrane association. Intriguingly, MiTMAB very profoundly inhibited M_2_ endocytosis to almost basal levels (Figure 6A,B). This degree of inhibition was very similar to that observed for the R399A dynamin-2 mutant, which is not capable of associating with membranes. Thus, our data imply that although dynamin GTPase activity appears to be largely dispensable for M_2_ endocytosis, dynamin as such is required and needs to be associated with membranes in order to facilitate M_2_ endocytosis.

### 2.2. Discussion

The mAChRs are rapidly internalized upon stimulation with an agonist, and the endocytosis of the M_1_, M_3_ and M_5_ receptor subtypes has previously been shown to be clearly clathrin- and dynamin- dependent [4,6]. However, M_2_ and M_4_ receptors evidently exhibit differential regulation of their uptake. Only recently, a novel mechanism, called FEME, which is clathrin independent, but dynamin-dependent, was identified as an endocytic pathway that mediates M_4_ receptor internalization [7]. In contrast, the mechanisms of endocytosis of the M_2_ receptor have remained less conclusive, although they have been addressed by several studies in the past. In particular, the involvement of clathrin and dynamin in M_2_ uptake has produced inconsistent results. We have here especially focused on dissecting the role of these two proteins, whereas other factors affecting M_2_ trafficking, such as β-arrestins and the small GTPase Arf6, were not a subject of the present study.

We here could show that uptake of the M_2_ receptor from the plasma membrane is clearly clathrin dependent in HEK 293T cells, whereas flotillin-mediated endocytosis, which is likely to involve membrane rafts, was excluded as a mechanism of M_2_ uptake. Several studies carried out in the past have concluded that M_2_ endocytosis is clathrin-independent. Many of these findings were based on expression of the dominant-negative clathrin hub mutant that showed no effect on M_2_ uptake upon agonist stimulation [6,19,24]. However, we here show that upon depletion of CHC, M_2_ endocytosis is profoundly impaired, demonstrating that clathrin is required for this process. We observed an inhibition of M_2_ endocytosis by about 60%, which correlated with the degree of CHC knockdown.

Interestingly, upon expression of the clathrin hub mutant, we could not detect any significant reduction in M_2_ endocytosis (data not shown), similarly to previous studies [6,19,24]. However, this might be due to an insufficient capacity of the hub mutant to impair clathrin function in the presence of the endogenous, WT CHC in these cells. On the other hand, endocytosis of M_1_ and M_3_ receptors is efficiently inhibited by hub expression in HEK293 cells [4]. The CHC hub mutant is a truncated form of CHC, comprising only the C-terminal part [24,37]. This fragment still undergoes trimerization and associates with the clathrin light chains. The resulting smaller clathrin triskelions are even able to form a lattice that exhibits less curvature and is open-ended in contrast to the strongly curved and closed polyhedron of WT clathrin [37]. Thus, it is possible that a mixture of the endogenous CHC and the exogenous hub mutant are capable of forming a mixed, mildly-impaired lattice that supports M_2_ endocytosis in HEK 293T cells. However, non-expression of CHC profoundly impairs M_2_ uptake, demonstrating that the agonist-induced endocytosis of the M_2_ receptor takes place by means of a clathrin-dependent endocytosis mechanism. However, in the case of the M_2_ receptor, the clathrin-dependent endocytosis may exhibit some atypical features that make it less sensitive to hub expression (see below).

As with clathrin, contradictory data have previously been obtained for the role of dynamin in M_2_ endocytosis. When the dominant-negative K44A dynamin mutant was expressed, it showed no effect on M_2_ uptake after CCh treatment, and a role for dynamin was thus excluded [4,9,12]. However, this may be cell-type dependent, as the expression of K44A dynamin in COS-7 cells impairs M_2_ uptake [4]. On the other hand, dynamin-1 mutants N272 and K535M substantially inhibited M_2_ endocytosis [22]. This is highly intriguing, as our data here also implicate that depending on how dynamin function is impaired by these mutations, the effect of the mutants on cargo endocytosis is very different. While the K44A mutant was not capable of inhibiting M_2_ uptake, consistent with previous findings [4,19], impairment of dynamin-2 GTPase activity by the T65A mutation only modestly reduced M_2_ uptake upon CCh stimulation. Similarly, chemical inhibition of dynamin GTPase activity by Dynasore produced only a minor effect on M_2_ endocytosis, which even appeared to be due to the known effect of Dynasore on the actin cytoskeleton [36] and not dynamin-2 inhibition *per se*. On the other hand, mutations and inhibitors that affect the membrane association of dynamin-2 (R399A and MiTMAB in the present study, K535M in [22]) substantially inhibited M_2_ endocytosis. Therefore, the data from the present and from previous studies would indicate that membrane association of dynamin-2 is required, whereas the GTPase activity as such is not a prerequisite for efficient M_2_ endocytosis to occur. However, care needs to be taken when drawing conclusions on the exact role of dynamin in cargo endocytosis, as this appears to be cell type specific, as shown by different effects of K44A dynamin in different cells [4,9,12], and may depend on the expression of specific accessory proteins in the cell line used. The use of a single or a few different cell lines is clearly a limitation of all studies, including ours, carried out so far. Thus, these studies should be expanded in the future to more physiologically relevant systems, such as primary cells, in order to better tackle their biological significance.

The idea that depending on the cargo to be endocytosed, clathrin-mediated endocytosis may exhibit differential requirements for the endocytosis machinery involved, e.g., dynamin, is highly provocative. However, this hypothesis is supported not only by the data obtained in the present study, but also by the studies cited above. Importantly, it has become clear that cargo may have an important role in the regulation of the fine-tuning and dynamics of coated pits [38]. Thus, not all clathrin-coated structures are alike and may show differences in terms of, e.g., accessory proteins involved in the endocytosis of specific cargo, e.g., GPCRs [38]. This is also supported by the findings demonstrating that although endocytosis of distinct cargo molecules is saturatable, it appears not always to be competitive between the cargo molecules [39,40,41]. Furthermore, differences in the composition and a functional specialization of clathrin-coated structures have been suggested by numerous studies [42,43,44]. Thus, it is possible that a subset of clathrin-coated pits involved in M_2_ endocytosis may exhibit an untypical and differential requirement for dynamin GTPase activity. At present, we are not able to provide a detailed explanation for how dynamin might be able to support vesicle fission in the absence of its GTPase activity. However, recent findings have shown that even the GTPase-deficient mutants of dynamin, including K44A, are able to promote membrane constriction in liposomes to a 3.7-nm inner diameter. As the theoretical limit for a spontaneous formation of a hemifission intermediate of a lipid bilayer is 4 nm, it may be possible to obtain fission without dynamin GTPase activity [45,46]. In light of these intriguing perspectives, further studies should be carried out to dissect the requirements for dynamin activity during clathrin-mediated endocytosis of specific cargo molecules that utilize different subsets of clathrin-coated pits.

## 3. Experimental Section

### 3.1. Cell Culture, Transfection and siRNA Knockdown

HEK 293T cells were cultured in Dulbecco’s Modified Eagle’s Medium (DMEM), high glucose, supplemented with 10% fetal calf serum and 1% penicillin/streptomycin (all from Life Technologies, Darmstadt, Germany). The cells were grown in a humidified incubator at 37 °C and 8% CO_2_. Cells were transiently transfected with plasmids using MACSfectin Reagent (Miltenyi Biotec, Bergisch Gladbach, Germany) according to the manufacturer’s instructions. Experiments were performed 24 h post-transfection. Transient depletion of CHC and flotillins was performed using MACSfectin Reagent according to the manufacturer’s instruction and siRNA obtained from Life Technologies (StealthTM siRNA system), as described in [47]. After 48 h, cells were additionally transfected with M_2_-EGFP or M_2_-dsRed DNA. The experiments were performed 72 h post-siRNA transfection.

### 3.2. Constructs and Plasmid Cloning

The dynamin-2-GFP fusion constructs (WT, K44A, T65A, R399A) have been described previously [47]. Human M_2_ was cloned into a pEGFP-N1 and a pDsRed-Monomeric-N1 vector (Clontech, Mountain View, CA, USA). The tags were added in the immediate C-terminus of the receptor, which results in a functional receptor molecule and does not impair its trafficking [10,17].

### 3.3. Antibodies

Mouse monoclonal antibodies against CHC, flotillin-1 and flotillin-2 were purchased from BD Biosciences (Franklin Lakes, NJ, USA). A polyclonal antibody against flotillin-2 (C-terminal) was obtained from Sigma-Aldrich (Taufkirchen, Germany). The mouse monoclonal antibody against GAPDH was obtained from Abcam (Cambridge, UK). Secondary antibodies against mouse and rabbit IgGs coupled to Cy3 or Cy5 were purchased from Dianova (Hamburg, Germany). Horseradish peroxidase-coupled secondary goat antibodies against mouse IgG for the detection of Western blots were purchased from Dako (Glostrup, Denmark). Actin filaments were stained with Alexa Fluor 594-coupled phalloidin (Life Technologies).

### 3.4. Immunofluorescence

HEK 293T cells were grown on coverslips and transfected with M_2_-EGFP or M_2_-DsRed constructs and, when indicated, with siRNA. Dynamin-2 constructs were cotransfected with M_2_-DsRed. Cells were starved in serum-free medium for 18 h and stimulated with 1 mM CCh (Sigma-Aldrich) for 15 min at 37 °C. Cells were fixed with methanol for 10 min at 4 °C or 10 min at room temperature with 4% paraformaldehyde (in 80 mM PIPES, 2 mM MgCl_2_ and 5 mM EGTA). Unspecific protein binding sites were blocked using 1% bovine serum albumin. Cells were labeled with primary antibodies or 10 µg/mL phalloidin. Secondary antibodies were incubated together with 4’,6-diamidino-2-phenylindole (DAPI, Merck, Darmstadt, Germany) for counterstaining of the nuclei. Cells were mounted in Fluoromount (Sigma-Aldrich) supplemented with 50 mg/mL 1,4-diazadicyclo(2,2,2)octane (Fluka, Neu-Ulm, Germany). The specimens were analyzed using a Zeiss LSM710 Confocal Laser Scanning Microscope (Carl Zeiss, Jena, Germany).

### 3.5. Inhibitor Treatment

M_2_-transfected HEK 293T cells were treated with 80 µM dynasore (Sigma-Aldrich) or DMSO as a negative control for 2 h prior to CCh stimulation, whereas MiTMAB (30 µM, Abcam) was added 30 min prior to CCh stimulation. CCh stimulation was performed in the presence of the inhibitors or the relevant control. To monitor the efficiency of the inhibitors, uptake of Alexa Fluor 546-labeled transferrin (Tfn546, Life Technologies) was used. For this, cells pretreated with the inhibitor were incubated with 10 µg/mL Tfn546 for 15 min at 37 °C. The surface-bound Tfn546 was removed with a stripping buffer (300 mM NaCl, 200 mM acetic acid, pH 2.9), after which the cells were fixed. The actin cytoskeleton was disintegrated with cytochalasin D (Sigma-Aldrich) and latrunculin A (Cayman Chemical, Ann Arbor, MI, USA). Pretreatment with cytochalasin D (3.9 nM) was performed for 2 h, whereas latrunculin A (1 µM) was added 45 min prior to CCh stimulation.

### 3.6. Cell Lysis, Gel Electrophoresis and Western Blot

Cells were lysed in 50 mM Tris (pH 7.4), 150 mM NaCl, 2 mM EDTA and 1% Nonidet P-40 supplemented with protease inhibitor cocktail (Sigma-Aldrich). Protein concentration was measured with the protein assay reagent of Bio-Rad (Munich, Germany). Equal protein amounts were analyzed by SDS-PAGE and Western blot.

### 3.7. Statistical Analysis

All experiments were performed at least three times independently of each other. To quantify the number of cells showing internalization of the M_2_ receptor, at least 100 cells were counted per condition. The percentage of cells showing translocation of the receptor from the plasma membrane to intracellular vesicles was calculated and used for the data analysis. Data are shown as the mean ± SD. For statistical comparison, two-way analysis of variance (ANOVA) was employed using GraphPad Prism 5 (GraphPad Software, La Jolla, CA, USA). Values of *p* < 0.05 were considered significant (*), whereas values of *p* < 0.01 and *p* < 0.001 were defined as very significant (**) and highly significant (***), respectively.
